# Anhydrous trifluoroacetic acid pretreatment converts insoluble polyglutamine peptides to soluble monomers

**DOI:** 10.1016/j.dib.2015.11.007

**Published:** 2015-11-14

**Authors:** Gunasekhar Burra, Ashwani Kumar Thakur

**Affiliations:** Department of Biological Sciences and Bioengineering, Indian Institute of Technology Kanpur, 208016, UP, India

## Abstract

The data provided in this article are related to the research article entitled “Unaided trifluoroacetic acid pretreatment solubilizes polyglutamine (polyGln) peptides and retains their biophysical properties of aggregation” by Burra and Thakur (in press) [1]. This research article reports data from size exclusion chromatography (SEC), reversed phase-high performance liquid chromatography (RP-HPLC) and mass spectrometry (MS) assays. This data show that trifluoroacetic acid (TFA) has the ability to convert insoluble polyGln peptides to soluble monomers. The data also clarify the possibility of trifluoroacetylation modification caused due to TFA. We hope the data presented here will enhance the understanding of polyGln disaggregation and solubilization. For more insightful and useful discussions, see the research article published in Analytical Biochemistry: Methods in the Biological Sciences (Burra and Thakur, in press [1]).

**Specifications Table**

**Table:** 

Subject area	*Biology*
More specific subject area	*Protein aggregation*
Type of data	*Table, text file and figure*
How data was acquired	*Size exclusion chromatography, RP-HPLC and mass spectrometry*
Data format	*Analyzed*
Experimental factors	*Polyglutamine containing peptides were disaggregated and solubilized by TFA pretreatment*
Experimental features	*SEC chromatogram, RP-HPLC chromatogram and LC-MS/MS spectra were obtained to check the state of peptide in solution*
Data source location	*Kanpur, India*
Data accessibility	*With this article*

**Value of the data**

• The data validate the information provided in the research article by Burra and Thakur [1].
• The data provide the detailed information related to disaggregation and solubilization of polyglutamine peptides.
• SEC data reveal the conversion of otherwise insoluble peptides to soluble monomers.
• RP-HPLC and LC-MS/MS data indicate the possibility of minor trifluoroacetylation modification of polyGln peptides.

## 1 Experimental design and data

### 1.1 Experimental design

A disaggregation condition, wherein the polyGln containing peptides were pretreated with anhydrous TFA was tested. The soluble fraction of peptide was subjected to SEC to check the conversion of otherwise insoluble peptides to soluble monomers through disaggregation. The chemical integrity of the peptides were checked by using RP-HPLC and mass spectrometry. The data was analyzed and compared to that of the existing TFA/HFIP method of polyGln disaggregation.

### 1.2 Physico-chemical properties of TFA and HFIP

The PubChem compound and ChemBank databases for the physical and chemical properties of TFA (CID: 6422) and HFIP (CID: 13529) indicate that they possess similarity with respect to their H-bond donor and acceptor abilities (Table 1). Hence, they may act in a similar manner while breaking or forming H-bonds when used for disaggregating insoluble peptides.

### 1.3 Size exclusion chromatography (SEC) data

**Fig. 1:**
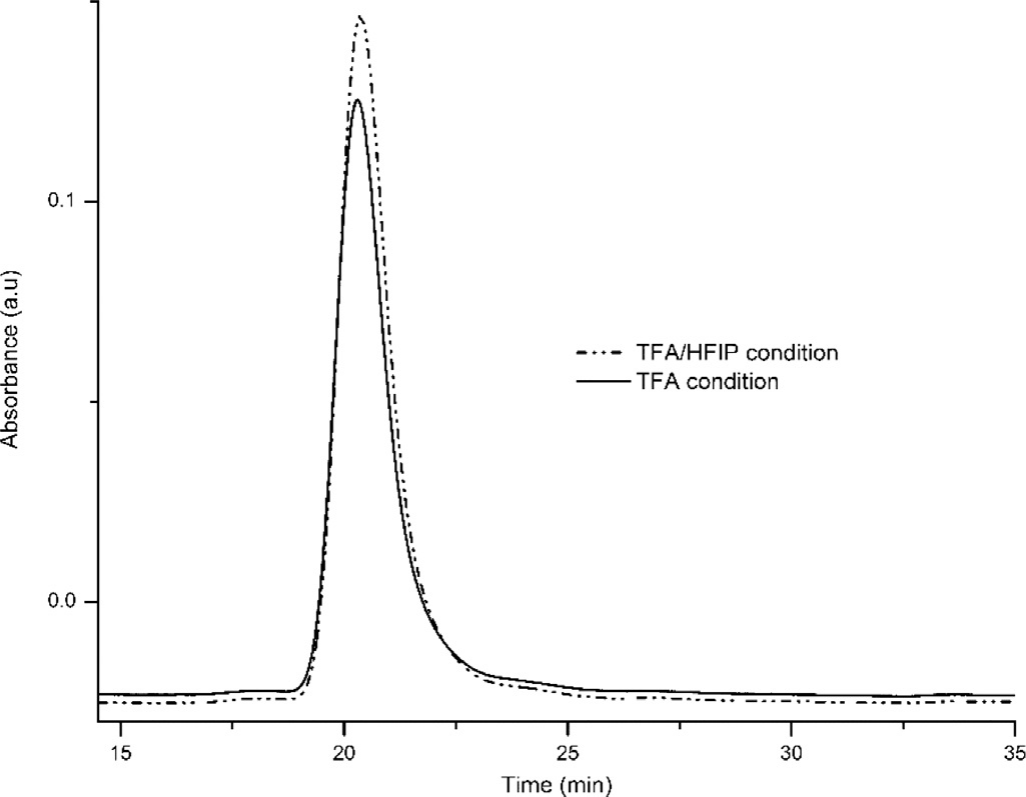
Size exclusion chromatograms of PGQ_9_I peptide in water-TFA (pH 3.0).

In Fig. 1, size exclusion chromatogram suggests monomeric state of peptide in solution after disaggregation and solubilization. The overlapping of TFA condition chromatogram with that of TFA/HFIP condition further suggests that they work in a similar manner for disaggregating polyGln peptides. Also, the absence of peaks in between the void-volume (~14.5 min) and the peak indicating monomer (~21 min) indicates the absence of any other higher order structures. This data is in accordance with previously published data on polyGln peptides of different length [2], [3], [4].

### 1.4 Analytical RP-HPLC data

**Fig. 2:**
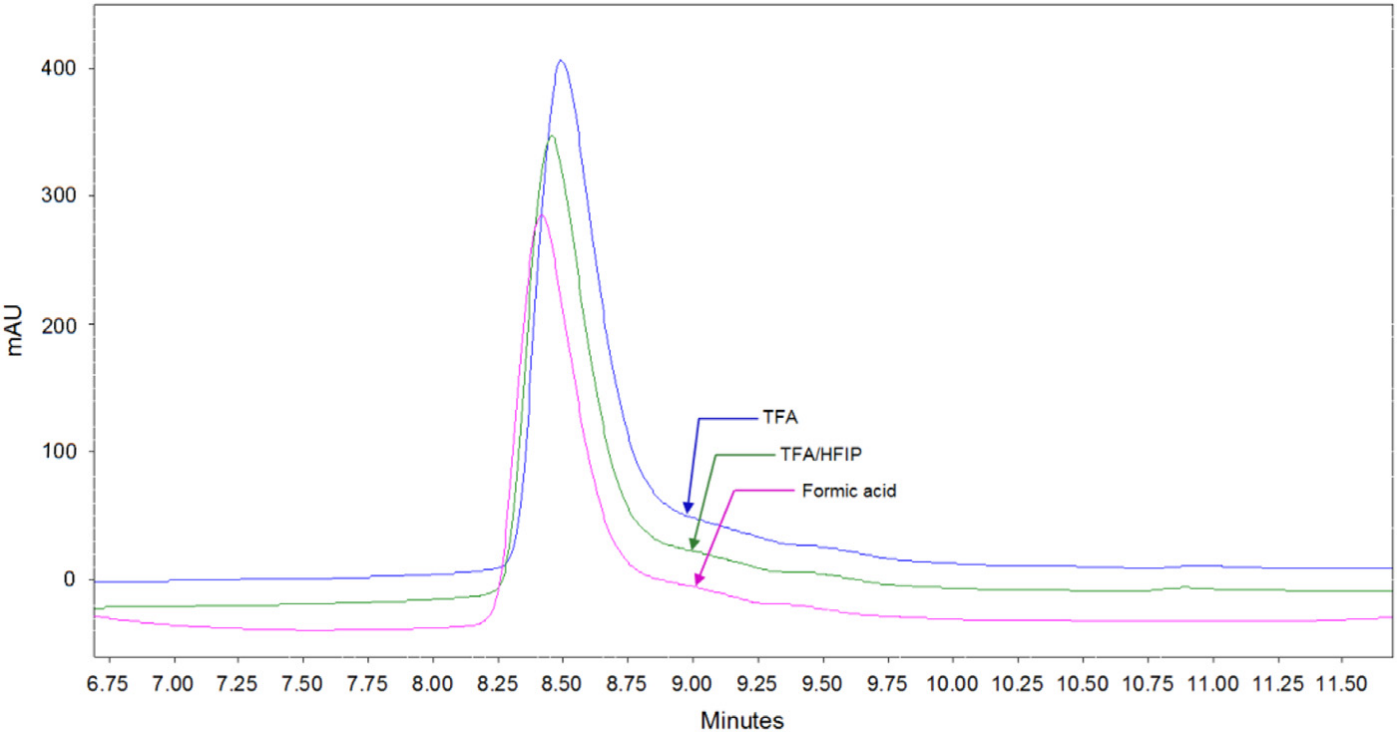
RP-HPLC chromatograms of PGQ_9_I peptide disaggregated in TFA, TFA/HFIP and formic acid conditions. The data is highlighted at the end of each peak to show the possible trifluoroacetylation modification in peptides.

Trifluoroacetylation of amino groups in peptides is a possible modification associated with the usage of TFA. This was analyzed by comparing the RP-HPLC chromatograms of PGQ_9_I peptide under three disaggregation conditions. The data show the presence of a small shoulder at the end of each chromatogram hinting the possible modification (Fig. 2). As polyGln peptides are not soluble in other solvents including water, the chromatogram before disaggregation was not obtained for comparison. However, formic acid was used to solubilize it before injecting in RP-HPLC.

### 1.5 Mass spectrometry data

**Fig. 3:**
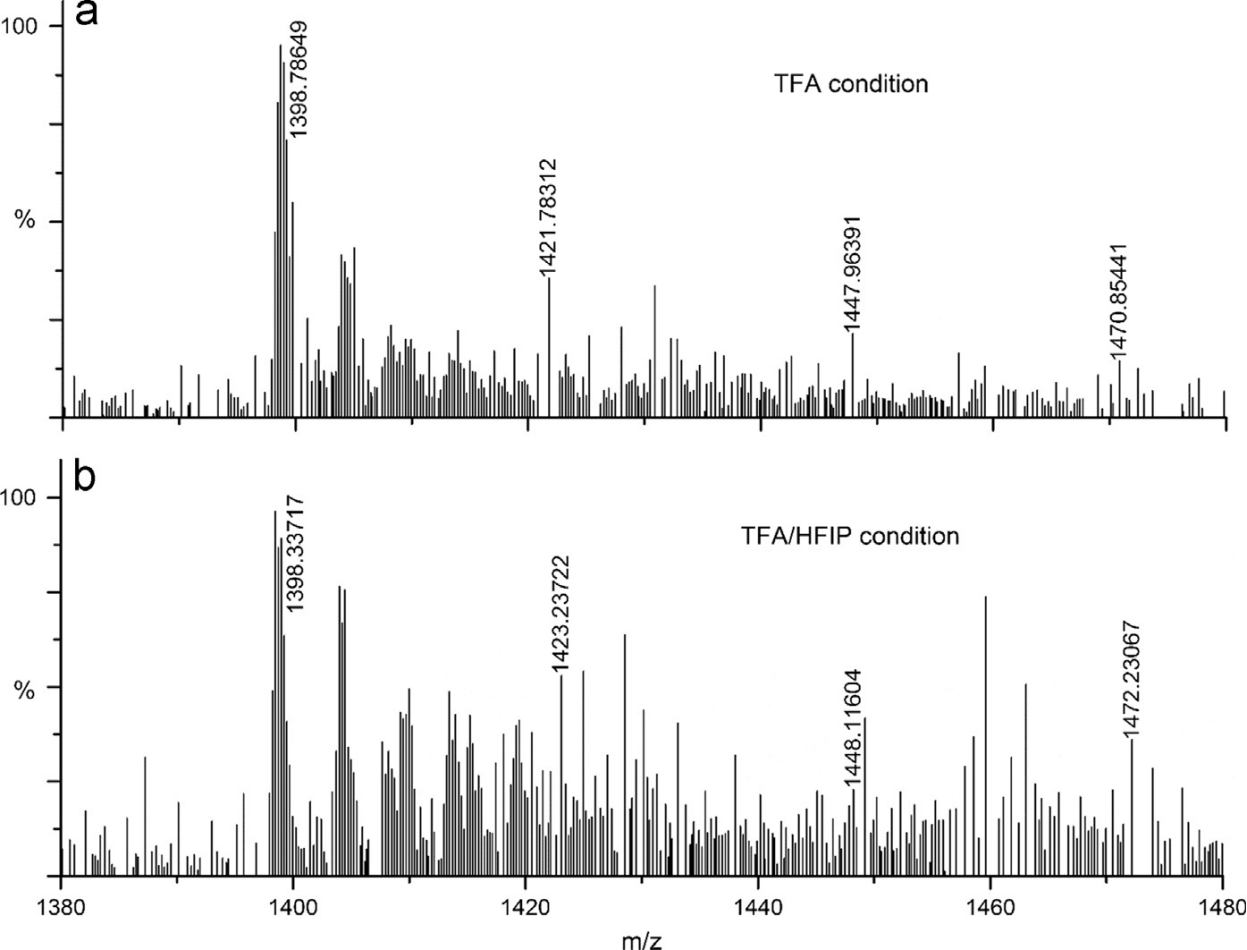
ESI-mass spectrometry data of PGQ_9_I peptide disaggregated under TFA and TFA/HFIP conditions. (a) and (b) indicates the isotopic mass (*m*/*z*) distribution of the peptide under TFA and TFA/HFIP disaggregation condition, respectively. The data shows isotopic mass distribution of the peptide (*m*/*z*, 1398) and the presence of few peptide ions with trifluoroacetylation modification (*m*/*z*, 1423, 1448 and 1471). The expected average isotopic mass of the peptide is ~5594 Da (M+4H)^+^.

The mass spectrometry data suggests that the majority of the peptide is pure and lacks any modification due to trifluoroacetylation in both the disaggregation conditions. However, the presence of modest amount of ions with possible trifluoroacetylation modification (*m*/*z*, 1423, 1448 and 1471) was observed when compared to the expected ions (*m*/*z*, 1398) (Fig. 3). This data indicates occurrence of modification in both the disaggregation conditions. It is difficult to provide any comparative analysis for the MS data before disaggregation because the polyGln peptides are not soluble in water and other buffer conditions without the pretreatment by TFA and HFIP.

### 1.6 Comparison of processing cost of disaggregation under TFA and TFA/HFIP methods

As stated earlier, the cost of HFIP per milliliter (product 105228-500G, Sigma-Aldrich India) is more than four times as compared to TFA per milliliter (product T6508-500ML, Sigma-Aldrich India). Here we compare the approximate cost of processing of 10 mg of NT_17_Q_35_P_10_ peptide [1]. For this, we used the approximate cost per milliliter of TFA (0.56 USD) and HFIP (2.5 USD) as quoted by Sigma-Aldrich India. The total volumes of TFA and HFIP required to solubilize at 0.1 mg/ml concentration though TFA/HFIP method are 50 ml each. This leads to a processing cost of 153 USD. Whereas if proposed TFA method is used, the total processing cost would be only 56 USD leading to a saving of ~97 USD. This supports that the proposed method is economical as compared to the existing method of polyGln disaggregation and solubilization. The above calculations were made as per quoted by Sigma-Aldrich, India and hence may subject to vary depending on the quality of the product, vendor and sizes of orders.

## 2 Materials and methods

### 2.1 Materials

PGQ_9_I peptide (K_2_-Q_9_-PG-Q_4_-I-Q_4_-PG-Q_9_-PG-Q_9_-K_2_) was purchased in crude from the Keck Biotechnology Center, Yale University. TFA, HFIP and formic acid were purchased from Sigma-Aldrich. Chromatography grade acetonitrile and sodium azide was from Merck and SDfine chemicals Ltd., respectively. Phosphate-buffered saline (PBS) was prepared as per the standard procedure described in Cold Spring Harbor protocols.

### 2.2 Disaggregation and solubilization of PGQ_9_I peptide

PGQ_9_I peptide was disaggregated under two different conditions: 1) overnight incubation in 1:1 ratio of TFA/HFIP at 0.5 mg/ml peptide concentration [5], [6] and 2) overnight incubation in only TFA at 0.5 mg/ml concentration. After overnight incubation, the volatile solvents were evaporated using a gentle stream of nitrogen gas, producing a thin peptide film on to the glass walls. To further ensure the complete removal of any residual TFA or TFA/HFIP, they were subjected to drying under vacuum in a desiccator for about 1 h. The peptide film was then solubilized in water-TFA (pH 3.0) and subjected to gentle swirling to ensure complete solubility [5], [6]. The peptide was also solubilized by incubating for 1 h in formic acid for analytical RP-HPLC study. This was then diluted to 20% formic acid by adding Milli-Q water just before injecting in RP-HPLC.

### 2.3 Analytical size-exclusion chromatography

PGQ_9_I peptide disaggregated under TFA and TFA/HFIP conditions was solubilized in water-TFA (~72 µM) for SEC analysis [4]. 50 µl was injected into SuperdexTM peptide 10/300GL (GE healthcare) SEC column connected to a Bio-Rad (Biologic Duoflow) chromatography system. The flow rate was maintained at 0.5 ml/min and the peptide was detected at 214 nm under a constant pressure of ~ 80 psi.

### 2.4 Analytical RP-HPLC chromatography

The PGQ_9_I peptide solubilized under three different conditions was injected into Agilent eclipse plus C_18_ column (4.6×100 mm^2^) connected to Agilent, 1260 Infinity Quaternary LC System. The solvent system used was water and acetonitrile, containing 0.05% (v/v) TFA [7], [8]. A linear gradient wherein the acetonitrile percent was increased from 2% to 34% in 9 min was used for elution of the peptide.

### 2.5 Mass spectrometry

PGQ_9_I peptide pretreated with TFA and TFA/HFIP was solubilized in water-TFA (pH 3.0). 5 µl of each sample was injected into high resolution time-of-flight mass analyzer (TOF) with an electrospray ionization (ESI) source of Waters Q-TOF premier HAB 213. The *m*/*z* spectra was recorded within a mass range of 50–3500. Data was plotted using OriginPro 8.5 version data analysis software. The different molecular ionization states of peptide were processed to identify any associated peptide modification.

## Figures and Tables

**Table 1:** Some important physical and chemical properties of TFA and HFIP.

**Property**	**TFA**	**HFIP**
Molecular weight (g/mol)	114.02	168.05
Molecular formula	C_2_HF_3_O_2_	C_3_H_2_F_6_O
Density (g/cm3)	1.49	1.60
Boiling point (°C)	72.4	58.2
H-bond donors	1	1
H-bond acceptors	5	7
pKa	0.3	9.3
